# How Do German General Practitioners Assess Medical Specialist Care Needs of Nursing Home Residents? Results of a Postal Survey in North-Western Germany

**DOI:** 10.3390/ijerph17197126

**Published:** 2020-09-29

**Authors:** Ove Spreckelsen, Guido Schmiemann, Alexander Maximilian Fassmer, Bettina Engel, Falk Hoffmann, Michael Hermann Freitag

**Affiliations:** 1 Division of General Practice, Department of Health Services Research, Carl von Ossietzky University of Oldenburg, 26129 Oldenburg, Germany; bettina.engel@uol.de (B.E.); michael.freitag@uol.de (M.H.F.); 2 Department of Health Services Research, Institute for Public Health and Nursing Science, University of Bremen, 28359 Bremen, Germany; schmiemann@uni-bremen.de; 3 Institute for General Practice, Hannover Medical School, 30625 Hannover, Germany; 4 Division of Outpatient Care and Pharmacoepidemiology, Department of Health Services Research, Carl von Ossietzky University of Oldenburg, 26129 Oldenburg, Germany; alexander.fassmer@uol.de (A.M.F.); falk.hoffmann@uol.de (F.H.)

**Keywords:** nursing home residents, primary care, specialist care, health services research, survey, Germany, coordination of care

## Abstract

The frequency of contacts of nursing home residents with medical specialists is lower compared to the general population of the same age group in Germany. The aim of this study was to assess general practitioners’ (GPs) views on specialist care needs of nursing home residents, on questions of qualification and care coordination. A cross-sectional study was conducted with a postal questionnaire among a representative sample of 1121 GPs in north-western Germany in 2018. The perceptions of GPs about the relative importance of the type of specialist care that is required in nursing homes was assessed on a five-point Likert scale (0 = very low to 4 = very high). A total of 375 GPs (response 33.5%; mean age 54.4 years; 57.6% male) participated in the survey. GPs assessed care needs as highest for neurologists and psychiatrists (68.7%) and lowest for gynecologists (6.5%). Almost all respondents (96.2%) strongly agreed that medical care for nursing home residents should be coordinated by GPs and that GPs should initiate the referral for further specialist care when required (87.5%). A minority (25.7%) agreed that quality of medical care would improve when care for a nursing home was provided by only one GP practice. GPs perceive the needs of nursing home residents for specialist care as high only in relation to care by neurologists and psychiatrists. GPs consider their own coordination function for medical care in nursing homes as very important.

## 1. Introduction

The provision of health care for people living in long-term care facilities is a challenge for health systems. In Germany, there are currently about 818,000 residents living in 11,200 nursing homes [1]. The average health status of nursing home residents is worse than the non-institutionalized counterparts of the same age with a high proportion of cognitive deficits, mobility constraints, and an overall high disease burden [2,3,4,5,6].

While a poor health status corresponds with high demands of medical care, in health research and policy it is subject to discussion as to whether this demand is adequately met by ambulatory medical specialists in nursing homes in Germany [4,7,8]. Among nursing home residents higher care dependency was found to be associated with a lower probability to receive care by a medical specialist [9]. Research from other countries shows that challenges to the provision of medical specialist care for nursing home residents are not restricted to the German context [10]. Apart from neurologists and psychiatrists, an under-supply of specialist care is assumed as contact frequencies of nursing home residents with medical specialists are lower compared to the general population of the same age [11]. However, some authors also claim an under-supply of care by neurologists and psychiatrists given the high proportion of cognitive deficits and neuro-psychological disorders in that patient-group [4,11]. Inadequate specialist care can lead to complications, a reduced quality of life or even higher mortality [12,13,14]. Further problems might be the contribution to high rates of potentially avoidable hospital admissions [15] or an oversupply of psychotropic medications [4]. However, there is no consensus about this and only a small evidence base on underlying care needs for medical specialists by nursing homes residents in Germany [16].

Health care for nursing homes residents can be scattered with uncoordinated and sometimes conflicting therapeutic approaches between specialists and general practitioners (GPs), a lack of knowledge of therapeutic decisions between involved physicians as well as nursing staff, and coexisting problems of over- and under-supply of medical care [4,7]. As with any other citizens, nursing home residents are free to choose their GP. The transition to a nursing home does not automatically lead to a change of the GP or other physicians but might do so, for example, if the distances between the practice and the nursing home are too far. There are no regulations regarding which and how many GPs and medical specialist provide care for the residents of a nursing home and only a minority of facilities have formalized arrangements for cooperation between GPs and nursing home staff [17].

Medical care in nursing homes is mainly provided by GPs as most nursing home residents are regularly in contact with a GP [4,8]. Although the transfer for a practice visit is mostly covered by the insurance, i.e., due to mobility constraints and cognitive deficits of the residents, a substantial proportion of care is provided through nursing home visits by physicians. Medical specialists conduct fewer visits to nursing homes than GPs [18]. In contrast to the more disease-oriented approach by specialists, GPs ideally view the patient holistically and know of any relevant health problem, medication, and therapeutic preference. 

Medical care for nursing home residents requires knowledge and skills in palliative and geriatric medicine [19]. However, there are no formal regulations in Germany to provide medical care for nursing home residents other than the qualification as a GP. The GP residency consists of at least five years with a mandatory one-and-a-half to two years in general practice and rotations through other specialties including internal medicine both in outpatient and in-hospital care [20]. Alternatively, a fully in-hospital residency of five years in internal medicine allows practice as a GP. For both pathways there are minimal requirements in terms of the number of procedures and types of patients treated [21], and it is possible to qualify as a GP with no specific focus on aged care. GPs are required to prove their participation in certified continuous medical education activities while they are free to choose the content and type [22]. Palliative and geriatric medicine are sub-specializations that GPs could additionally qualify in. However, most qualified geriatricians work in hospitals and rather seldom in nursing homes. Equally, in most European countries there are no widely accepted standards for care provision by GPs in nursing homes [5]. Some evidence from the US suggests higher quality of care by physicians specifically qualified for care in nursing homes [23].

To address these gaps in knowledge about GP views on specialist care, the organization of care and necessary qualifications, we wanted to know:

- How do GPs rate specialist care needs of nursing home residents in Germany?
- How do they view their coordination function, the appropriateness of their qualification for nursing home care, and the provision of service by only one practice?

## 2. Materials and Methods 

### 2.1. Study Design

In August 2018, a cross-sectional study among GPs and general internists working in primary care was conducted in the federal states of Bremen and Lower Saxony. A sample of 1221 participants was drawn from a list of the respective Associations of the Statutory Health Insurance Physicians (“Kassenärztliche Vereinigungen”; of a population of approximately *n* = 5500 GPs). Participants were invited by postal letter to complete a paper-based questionnaire and a reminder was sent after two weeks. All data were collected anonymously.

Sample size calculation followed a previous British survey of GPs about care in nursing homes [24]. To increase response to the postal survey several strategies shown by a Cochrane review were applied [25] including a short questionnaire, pre-paid return envelopes, and a follow-up contact. This questionnaire was part of the “HOspitalizations and eMERgency department visits of Nursing home residents” (HOMERN) project to assess frequencies and reasons for hospital transfers of nursing home residents [26].

### 2.2. Questionnaire

A multidisciplinary research team (with public health and GP background) developed the four-page questionnaire on medical care provision in nursing homes, hospital admissions and emergency department visits, and end-of-life care in nursing homes. This present study is based on the section related to views on medical specialist care needs, coordination of care and qualification of physicians.

First, we asked the participants whether they agreed that medical care for nursing homes residents should be coordinated by GPs and whether they agree that contacts to medical specialists should follow a GP referral (on a regular basis; both response categories binary “yes or no”). Medical specialist care needs of nursing homes residents were assessed by asking respondents to estimate the importance of the most common specialties working in ambulatory care, namely ophthalmologists, surgeons/orthopedists, dermatologists, gynecologists, otorhinolaryngologists, psychiatrists/neurologists, urologists, and dentists (all answers on a five-point Likert scale ranging from 0 = very low to 4 = very high). As most geriatricians provide care in hospitals and it is therefore uncommon to find geriatricians practicing in nursing homes, we did not include this specialization as a response category. Equally, palliative medicine is provided by GPs or other specialists (i.e., anesthesiologists) with a sub-specialization, we therefore did not treat palliative medicine as a separate specialization from GPs. Furthermore, GPs views on end-of-life and palliative care in nursing homes are published elsewhere [26]. Finally, we asked respondents to state the degree of agreement with the following statements: whether the residency was considered as a sufficient qualification for providing medical care for nursing homes residents and whether the quality of care might be better when only one GP practice provided care for all nursing homes residents in one nursing home. All answers were collected on the same Likert scale as above.

Additionally, sociodemographic characteristics of the GPs were requested: age, sex, number of years working as a GP, additional qualifications in palliative care and geriatrics, practice type (single practice, group practice/medical care center) and location (rural ≤20,000, semi-urban >20,000 to ≤100,000, urban >100,000 inhabitants), number of nursing homes visited and number of residents cared for. 

The questionnaire was piloted among and discussed with several GPs involved in medical care for nursing home residents before data collection was started. These GPs were not part of the research group and were from another region than the study area. The questionnaire was intended to obtain information from GPs of their assessment of the relative importance of different medical specialties for health care of nursing home residents on the health care system level. In the (cognitive) pretests this understanding of the items (aiming at health care needs of nursing home residents) was validated. An average low rating of health care needs of a particular specialty would therefore be interpreted as a lesser relative importance than an average higher rated specialty. In the pretests this understanding was not contradicted by the fact that any medical specialty could be of great importance for an individual nursing home resident for a specific health problem.

We also asked the participants to make a judgement about a clinical case scenario involving a hospital transfer of a nursing home resident, which will be separately analyzed.

### 2.3. Statistical Analysis

We computed descriptive measures for all answers and calculated frequencies for categorical data. Responses were compared between GPs with different sociodemographic characteristics (described above) using chi-square tests to assess statistical significance. We combined the answers ‘very high’ and ‘high’ as well as ‘very low’ and ‘low’ to one answer category, respectively. 

A statistically significant difference was stated for *p* < 0.05. All statistical analyses were performed using STATA for Windows version 14.1 (StataCorp LLC, College Station, Texas, USA) and SAS 9.4 (SAS Institute Inc., Cary, NC, USA).

This study received a waiver by the local medical ethics committee of the Carl von Ossietzky University of Oldenburg (no. 2018-080).

## 3. Results

### 3.1. Respondents

The response was 33.5% (375 of 1121 mailed questionnaires; Table 1). The participants’ mean age was 54.4 years (standard deviation (SD): 9.3; range: 33–84). The majority were male (57.6%), worked in group practices or medical care centers (67.0%) and worked in rural areas (52.3%). A total of 24.3% held additional qualifications in palliative care and 4.8% in geriatrics. The respondents cared on average for 46.8 (SD: 43.5; range: 0–360) patients in 4.1 (SD: 2.2; range: 0–15) nursing homes.

### 3.2. Opinions on Care Needs of Nursing Home Residents by Different Specialists

**Figure 1:**
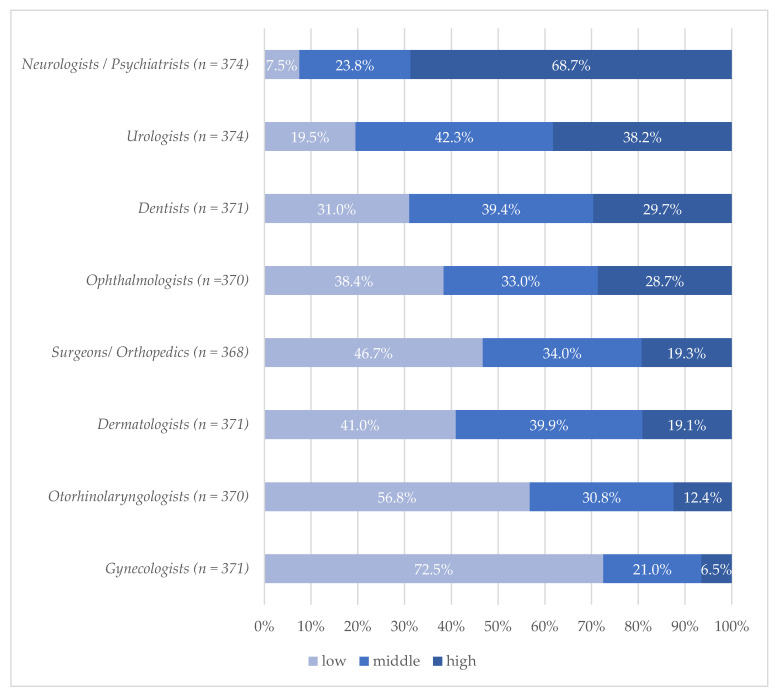
Medical specialist needs of nursing home residents assessed by general practitioners %/differences in *n* due to missing values. Question: “How do you assess the care needs (of nursing home residents) by different medical specialties?” (Original in German: “Wie schätzen Sie den Versorgungsbedarf durch folgende Arztgruppen ein?”).

The assessment of specialist care needs of nursing home residents was highest for neurologists and psychiatrists (68.7%; Figure 1). For all other specialties, only a minority rated the care needs as high with most prominent ratings for urologists (38.2%), dentists (29.7%), and ophthalmologists (28.7%). Lowest ratings were found for otorhinolaryngologists (12.4%) and gynecologists (6.5%). No relevant differences of the assessments were found by the sociodemographic characteristics of the respondents. 

### 3.3. Views on Coordination of Care and Referrals

Almost all respondents (96.2%) agreed with the statement that medical care for nursing home residents should be coordinated by GPs. Another large majority (87.5%) agreed that a referral from a GP should be a prerequisite for a consultation with a medical specialist for a patient from a nursing home if required (with exceptions for emergencies). Due to the strong agreements no further analyses for sociodemographic differences were conducted.

### 3.4. Opinions about Qualification and Single-Practice Policy 

The residency in general practice was viewed by the majority (59.4%) as sufficient for conducting primary care in nursing homes. None of the sociodemographic characteristics showed statistically significant differences.

Only a minority of 25.7% of the respondents agreed with the statement that the provision of primary care was of higher quality when provided by only one GP practice for one nursing home. Respondents with an additional qualification in palliative care showed somewhat higher agreements to the aforementioned statement than those without (agreement 36.3% vs. 22.3%, *p* = 0.028).

## 4. Discussion

The main finding of this study is that neurologists and psychiatrists are the only medical specialists for which German GPs perceive the care needs of nursing home residents as high. Care coordination and referrals to specialists by GPs are seen as important while no improvement of care is expected when care for one nursing home were provided by only one GP practice. A qualification as a GP is seen as sufficient for providing primary care in nursing homes.

The assessment that care needs of nursing homes residents are highest for neurologists and psychiatrists corresponds to the high burden of neuropsychiatric illnesses as cognitive deficits, gait disorders, or mood disorders that are known from national [4,11] as well as international studies [27,28]. Under-supply of specialist care is documented for patients with Parkinson’s Disease and with Alzheimer’s Disease [29]. However, approaches to care for nursing home residents might differ between specialists and GPs and might indicate a need for further research of the appropriateness towards medical care in this population. For example, GPs tend to be more in line with guideline recommendations to be more restrictive with antipsychotic treatments because of increased risks of cerebrovascular events and higher mortality among nursing home residents with dementia than neuropsychiatric specialists [30]. Further research would have to explore for which neuropsychiatric health problems or in which specific patient groups GPs need the assistance of neurologists or psychiatrists for adequate care provision.

Only a minority of respondents rated the care needs of nursing home residents as high for other specialties. These assessments contrasts with the high prevalence of health problems regarding oral health, swallowing difficulties, hearing impairments or incontinence [13,31,32] and possible subsequent health benefits of specialist consultations of dentists, ophthalmologists, or urologists [12,13,14]. Whether GPs assess the severity of the aforementioned health problems differently to specialists cannot be answered with our results. However, a more likely explanation is a different approach to care by GPs. GPs have to approach their patients holistically, acknowledge complex health problems and are—contrarily to hospital care—less focused on one leading disease [33]. Most nursing home residents are seen multiple times a year by a GP [4,8]. Therefore, we would assume that GPs are aware of the medical needs of the residents and might take care of various health problems that could also fall into the domain of different specialties. However, further research is needed to gain a more comprehensive picture of what type of care GPs actually provide for nursing home residents and in what situations GPs seek support from medical specialists. In addition, the high prevalence of multi-morbidity [3] of nursing homes residents is often not the focus for clinical guideline development aiming at a single disease. This may lead to uncertainties for GPs for the application of guidelines in nursing homes. The uncoordinated involvement of different specialties for one patient might lead to a number of problems including differences in therapeutic goals and polypharmacy [34]. End-of-life situations are examples in which GPs intentionally reduce the involvement of medical specialists for patients from nursing homes in favor of palliative approaches as the reduction of interventions and de-escalation of therapies [26]. 

Therefore, it is not surprising that German GPs prefer the coordination of medical care for nursing home residents by their own profession. However, a mandatory coordination of specialist care by GPs in nursing homes—while standard in many European countries—would require structural changes in the German health care system. Reforms aiming at better integration of health care services for nursing homes would need to build on shared goals and understanding of the involved professional groups.

The majority of respondents rejected a model of care that binds all patients from one nursing home to only one GP practice. Such an approach is proposed to facilitate the communication between nurses and physicians [4]. The stance of the respondents might be due to the assessment of the own care to be of high quality even when provided in nursing homes where patients are seen from GPs from different practices. Also, GPs might view the coordination of medical care by GPs much more important for improving medical care for nursing homes residents than models of care provision for one nursing home by a single practice that are uncommon in Germany or restricted to pilot projects. Furthermore, GPs might reject any health policy intervention that changes the current status quo of care provision for fears for loss of professional autonomy, especially when possibly involving the patient’s right to freely choose a physician to consult. 

While most respondents considered the GP residency as a sufficient qualification for care for nursing homes residents, a proportion of more than 40% did not agree with that statement. In contrast to the German model—where apart from the GP training no formal extra qualifications in palliative care or geriatric medicine are required—some international examples put a stronger and mandatory emphasis on specified geriatric knowledge applied to the special population of nursing home residents [19]. In the US, a specialization of generalist physicians in nursing home care is associated with quality improvements such as less use of antipsychotic medication or fewer indwelling bladder catheters [23]. The presentation and management of clinical problems such as malnutrition, infections or falls can differ between the community setting and among nursing home residents and additional problems of multiple drug interactions or dementia-related problems provide strong arguments for a specific focus on aged care [19]. A continuous relationship between a GP and a patient from a nursing home is characterized by the recognition of both multiple health problems and personal preferences of the patient or relatives. A stronger role of GPs in nursing home care might prevent some nursing home residents from avoidable hospital admissions [15]. There is some evidence that health care systems that ensure a greater continuity of care by employing salaried GPs in nursing homes are associated with an overall better quality of care [17].

With our study we are only roughly able to infer the degree to which GPs rely on specialist services to provide care for nursing homes residents. However, comparisons show that in nursing home care specialized GPs in the Netherlands do take over tasks that would fall into the domain of specialists in England [35], thus providing a possible starting point for a clearer definition of the scope of care provided by both GPs and medical specialists in nursing homes in Germany. Further studies would be needed to assess the needs of GPs of tailored continuous professional education programs as well as structured arrangements to consult specialists or other forms of specialist support. 

### Strengths and Limitations

The study is based on a survey of randomly selected GPs and met the expected response making it comparable to other studies [24]. 

The topic of medical care for nursing homes residents is of relevance to the respondents as almost all take care of nursing homes residents in at least one nursing home. However, some selection bias cannot be ruled out. For example, the proportion of GPs additionally qualified in palliative care is higher than the average. These respondents might be more interested in the topic than those without, as palliative situations appear frequently among nursing home residents and a part of our survey specifically focused on palliative care in nursing homes. Furthermore, it might be possible that in our sample those are over-represented who take care of a higher number of nursing home residents and possibly feel less dependent on the support of medical specialists. However, a validation of that assumption is not possible since it is not centrally registered to what degree GPs in Germany are on average involved in care in nursing homes. Also, ongoing political debates about reforming the care system during the data collection period might have spurred some special interests as well as induced reserved answer behavior concerning questions with possible implications for health policy.

The development of the questionnaire was based on prior research in the field and part of the larger HOMERN project to investigate frequencies and reasons for hospital transfers of nursing home residents. All questions and answers were carefully discussed and thoroughly developed in a multidisciplinary research team and piloted among several GPs with experiences in nursing homes. 

With our study we assessed the views of GPs on medical care needs of nursing homes residents. We did not ask specialists, whose points of view might differ. To narrow down which approaches are adequate in what situations and the specific tasks that would fall into the domain of a particular professional group would require discussions and mutual agreements between GPs and specialists involved in nursing home care. 

## 5. Conclusions

Our results underscore the importance of the adequate availability of neuropsychiatric care in nursing homes. However, and in contrast to discussions in health policy, GPs do not perceive the care needs of nursing home residents for all medical specialties as high. Further research would have to explore for which indications specialists are needed and which services can be provided by GPs, and similarly, when palliative approaches might be more beneficial. Subsequently, the consequences for qualification and continuing medical education especially in the fields of geriatrics and palliative care would have to be specified. Also, it would be valuable to increase research efforts to obtain reliable data on medical needs of nursing homes residents.

In terms of the organization of health care, GPs are strongly in favor of medical specialist care coordination conducted by GPs whereas they are reluctant towards binding care for nursing home to only one GP practice. As GPs are front-line personnel and main providers of medical care for nursing homes residents, the assessment of GPs perspectives on care might be valuable for the further development of health care for nursing home residents.

## Figures and Tables

**Table 1:** Characteristics of the respondents (*n* = 375).

Characteristics	
Age in years (*n* = 371) *	
Mean (±SD) Median (Interquartile range)	54.4 (±9.3) 54 (48–61)
Sex (*n* = 373) *	
Male	57.6%
Female	42.4%
Years as general practitioner (*n* = 373) *	
Mean (±SD) Median (Interquartile range)	18.0 (±10.8) 18 (9–25)
Practice type (*n* = 373) *	
Single practice	33.0%
Group practice/Medical care center	67.0%
Practice location (*n* = 373) *	
Rural (≤20,000 inhabitants)	52.3%
Semi-urban (>20,000 to ≤100,000 inhabitants)	25.2%
Urban (>100,000 inhabitants)	22.5%
Number of nursing homes visited (*n* =3 73) *	
Mean (±SD) Median (Interquartile range)	4.1 (±2.2) 4 (3–5)
Number of residents cared for (*n* = 367) *	
Mean (±SD) Median (Interquartile range)	46.8 (±43.5) 30 (20–60)
Trained in palliative care (*n* = 375) Trained in geriatric medicine (*n* = 375)	24.3% 4.8%

* Numbers differ due to missing values. SD = standard deviation.
